# Iranian Native Plants on Treatment of Cutaneous Leishmaniosis: A Narrative Review

**Published:** 2017

**Authors:** Elham MOGHADDAS, Ali KHAMESIPOUR, Mehdi MOHEBALI, Abdolmajid FATA

**Affiliations:** 1. Dept. of Parasitology and Mycology, School of Medicine, Mashhad University of Medical Sciences, Mashhad, Iran; 2. Center for Research and Training in Skin Diseases and Leprosy, Tehran University of Medical Sciences, Tehran, Iran; 3. Dept. of Parasitology and Mycology, School of Public Health, Tehran University of Medical Sciences, Tehran, Iran; 4. Skin Diseases and Cutaneous Leishmaniasis Research Center, School of Medicine, Mashhad University of Medical Sciences, Mashhad, Iran

**Keywords:** Cutaneous leishmaniasis, Herbal medicine, Treatment, Iran

## Abstract

**Background:**

Chemotherapy still relies on the use of pentavalent antimonials, amphotericin B, paromomycin, miltefosin, and allopurinol. In this study, we explained about the native plant that grows in different regions of Iran and used as anti-leishmanial in Iran and even many other countries.

**Methods:**

This narrative review covers all information about local herbal medicine in Iran that used in treatment of cutaneous leishmaniasis in all the worlds, published in local and international journals from 1996 to 2015 using various databases including PubMed, SID, Google Scholar, Scopus, and Science Direct.

**Results:**

Overall, 150 articles in databases were identified. Many local plants grown in some places of Iran were used to treat this endemic disease.

**Conclusion:**

The cutaneous leishmaniasis is also a major health problem in Iran, especially in Mashhad (Northeast of Iran). Therefore, many patients seek for herbal therapy that is cheaper and readily available. This review provides information regarding plant that exists in Iran and exhibiting effects on anti-*Leishmania* activity. Among the anti-leishmanial mentioned in this review, most have never been tested for cytotoxicity and very few have been tested for in vivo activity.

## Introduction

Cutaneous leishmaniasis (CL) is an endemic and sometimes hyperendemic disease in Iran, where it constitutes health problems. About 20000 cases of the disease are reported from different areas of Iran annually (1, 2). During 20 years (1995–2014) 68958 cases of CL were diagnosed only at 5 health centers of Mashhad, the capital city of Khorasan-E-Razavi Province, Northeast Iran (3).

Since 50 years ago pentavalent antimonials are the most common effective medicine used for treatment of CL. Using this group of drugs, sometimes associated with problems such as different responses of patients, drug toxicity, resistance, high failure rate, long course of therapy, and relapse (4). Cardiac and renal insufficiency has been recorded as side effect of these compounds. Despite these flaws, pentavalent antimony is still the first choice of treatment for treatment of different forms of leishmaniasis (5).

In the early 1980s, some reports about untreated patients with these drugs were announced, but unfortunately, there is still no development in the production of novel antileishmanial drug (6). All disadvantages led to an interest in the study of traditional remedies as a source for the development of new chemotherapeutic medicine with better efficiency and less toxicity and side effect. Antiprotozoal herbal drugs are potential sources of alkaloids, alavonoids, phenylpropanoid, steroids, and terpenoids (1, 2). Several plants have been used for the treatment of parasitic disease (Table 1).

**Table 1:** Summary of Iranian local plant that used for the treatment of cutaneous leishmaniasis in the world

***Plant species***	***Family***	***Extracts or compounds***	***Cultured place***	***Ref***
*Achillea millefolium*	Asteraceae	Essential oil	All of Iran	(12, 16)
*Tanacetum parthenium*	Asteraceae	Plant powder	Kohgiluyeh and Boyer-Ahmad Province	(18)
		Hydroalcoholic extract		(19)
		Dichloromethane extract		(20)
*Carica papaya*	Caricaceae	Tyrosyl esters	Sistan baluchestan	(21)
		Ethanolic extract		(22)
*Ginkgo biloba*	Ginkgoaceae	Isoginkgetin	North of Iran	(25)
		Quercetin		(58)
*Nigella sativa*	Ranunculaceae	Thymoquinone	Centre of Iran	(28)
		Alcoholic extract		(29)
*Berberis vulgaris*	Berberidaceae	Alcoholic extract	South Khorasan	(33)
		Berberine		(32)
*Plantago psyllium*	*Plantaginaceae*	Powder	North of Iran	(36)
*Alo vera*	*Xanthorrhoeaceae*	Leaf exudate	South Iran	(37)
		Aloe-emodin ointment		(39)
*Plantago major*	*Plantaginaceae*	Leaves extract	Kurdistan province	(40, 41)
		Dried powder and boiled leaves		(59)
*Allium sativum*	*Amaryllidaceae*	Alcoholic extract	All of Iran	(43, 44, 48)
		Allicin		(45–47)
*Green tea*	*Theaceae*	Ethanolic extract	Northern Iran	(50)
		Dietary polyphenol (flavanol)		(51)
*Thymus vulgarish*	*Lamiaceae*	Hydroalcoholic extracts	Mountains of Iran	(52)
		Essential oil		(53)
		Hexane extract		(54)
*Salvia officinalis*	*Lamiaceae*	Methanolic extracts	Northern Iran	(55, 56)
*Artemisia sieberi*	*Asteraceae*	Alcoholic Essence	Desert areas of Kashan	(61)
		Aqueous extract		(63)
*Lawsonia inermis*	*Lythraceae*	Hydroalcoholic extracts	South of Iran	(66, 67)
*Cassia fistula*	Fabaceae	Fruits extracts	Northern Iran	(53, 55, 56)
		Boiled extract Hydroalcoholic extract		
*Seidlitzia Rosmarinus*	Chenpodiaceae	Hydroalcoholic extract	Desert areas of Iran	(57, 58)
*Euphorbia myrsinitis*	Euphorbiaceae	Soxhlet extracts	Sistan baluchestan	(59–69)
*Satureja khuzestanica*	Lamiaceae	Ethanolic and methanolic leaf extracts	Khuzestan and Lorestan provinces	(70–74)

The present study reviewed native Iranian herbal medicine for treatment of CL.

## Methods

This narrative review covers all information about local herbal medicine in Iran that used in treatment of CL, published in local and international journals from 1996 to 2015 using various databases including PubMed, SID, Google Scholar, Scopus, and Science Direct.

## Results

### *Achillea millefolium* (Common name: *yarrow*; Persian name: *bumadaran*)

*A. millefolium* is a flowering plant in the family Asteraceae grown in northern hemisphere in Europe, Asia and North America (7). Nineteen species of *A. millefolium* have been recorded from Iran. The juice and extract of this plant have been used as anti-inflammatory, antioxidant, antispasmodic, stomachic and antiseptic drug (8). The gel form is also used as a wound healing in traditional medicine (9). Recently, essential oil extracted from the leaves and flowers of *A. millefolium* has been studied against *Leishmania* promastigotes (10, 11). Oil extract of A. *millefolium* make morphological changes and size of this parasite. Other changes appeared on flagella and cell membrane structures that may lead to rupture of the plasma membrane, formation atypical vacuoles and myelin-like figures in promastigotes (10).

### *Tanacetum parthenium* (Common name: *feverfew*; Persian name: *babouneh*)

*T. parthenium* grows in all regions of Iran with different climates. The roots and rhizomes of this plant have been used in Iranian traditional medicine under the name of *Aqhovan*. The plant contains numerous pharmacological compounds but the active ingredients are present e.g. *Sesquiterpene lactones* and parthenolide. Flavonoid glycosides and pinenes are the other active ingredients of *Aqhovan.* Anticancer, anti-inflammatory, cardiotonic, antispasmodic, emmenagogue, and as an enema for worms are some pharmacologic charactristics. (12). Powder form inhibited the growth of *Leishmania* spp. (13, 14). “In vitro and in vivo antileishmanial activity of sesquiterpene lactone-rich dichloromethane fraction obtained from *Tanacetum parthenium*” (15).

### *Carica papaya* (Common name: *pawpaw*; Persian name: *kharboze- derakhti*)

*C. papaya* brought to south of Iran in the 1990s from American countries. It is a famous fruit in tropical region of Iran and is cultivated is widely increasing in these areas. Ascorbic acid, α-tocopherol, beta-carotene, vitamin B1, flavonoids, and niacin are natural important constituent of papaya (16). Tyrosyl lipophilic and ethanoic extract have been reported as leishmanicidal materials on *L. major* and *L. infantum* promastigotes (17). It is also known as accessible, nontoxic used prophylactic and cost effectiveness drug for leishmaniasis treatment (18).

### *Ginkgo biloba* (Common name: *maidenhair tree*; Persian name: *ginko*)

In traditional Chinese medicines, everyone knows Ginkgo. Recently it is cultivated in Iran. Arediterpene lactones and flavonoides are main components of this plant (19). Approximately 300 different forms were extracted from the leaves ranging 22% and 27% of flavonoid glycosides (20). Isoginkgetin and ginkgetin from *G. biloba* leaves have anti-*Leishmania* properties (21). One importance flavonoids in this plant is Quercetin, can be absorbed by humans. Quercetin caused mitochondrial dysfunction and parasite death of *L. amazonensis* promastigote *in vitro* (22).

### *Nigella sativa* (Common name: *Black Cumin*, *Black Caraway*, *Black Onion Seeds*, *Black Sesame Seeds*, *Musta Siemen*, *Grani Neri*, *Hei Zhong*; Persian name: *siah daneh*)

*N. sativa* is a common plant traditionally used in the Iranian pickles, bread and foods. The *Nigella sativa* is used as diuretic, carminative, vermifuge and lactagogue (23). Essential oil, methanolic extract, and thymoquinone of *N. sativa* were studied on murine macrophages infected with leishman bodies. Thymoquinone showed higher anti-*Leishmania* effect than other extracts (24). In another study, honey based extract of *N. sativa* with glucantime is more effective than glucantime alone in scar size and reduce dose of glucantime (*P*=0.002) (25).

### *Berberis vulgaris* (Common name: *Barberry, European Barberry*; Persian name: *zereshk*)

*B. vulgaris* is cultured in Europe and Asia since ancient time. It is well known in Iran and some other countries in the world. Leaf, root, bark, and fruit have been used for gastrointestinal, cardiovascular, respiratory, skin, renal and infectious diseases. For many years, Iranian people used barberry juice to prevent and treat of fatty liver and high blood pressure (26). Palmatine and particularly berberine are the main important components of this plant (27). Berberin was effective on *L. tropica* and *L. infantum,* in vitro. In one study, alcoholic extract of stems, leaves, and root bark has been used for treatment experimental leishmaniasis inoculated by *L. major* in murine model, successfully (28, 29).

### *Aloe vera* (Common name: *aloe*; Persian name: *Sabrezard*)

*A. vera* grows in southern part of Iran (Boushehr). Remedy of bowel diseases, itching, diabetes, stomach ulcers, asthma, depression, and constipation were reported from Aloe gel (30). *A. vera* leaf exudate has antileishmanial effect on *L. braziliensis*, *L. mexicana*, *L. tropica*, *L. major* and *L. infantum* promastigotes and *L. donovani* amastigotes (31). Fraction of *A. vera*, *Coriandrum sativum* and *Ricinus communis* on promastigotes and amastigotes of *L. infantum* were used*. A. vera* did not differ from pentamidine (*P*> 0.05) but *R. communi*s and *C. sativum* were more effective than *A. vera* on amastigotes in cell culture (32). Growth of *L. major* amastigotes in vivo and promastigotes in vitro were inhibited by Aloeemodin ointment (1, 8-dihydroxy-3-hydroxymethyl-anthraquinone) (33).

### *Plantago major* (Common name: *Birdseed, Broadleaf Plantain, Healing Blade, Hen-plant, White ManÕs Foot*; Persian name: *barhang*)

*Plantago* spp. is useful to treatment of disorders such as respiratory, wound healing, digestive organs, inflammation, reproductive system, cancer, and blood circulation (34). The genus *Plantago* comprises 16 species in Iran. Powder dried leaves with honey were used orally before breakfast to healing of ulcers and to treat *L*. *braziliensis* skin ulceration (35). In rural area of Brazil, bathing with boiled leaf extract and dried leaf powder is common to treatment cutaneous leishmaniasis due to *L. braziliensis* (36).

### *Allium sativum* (Common name: *garlic*; Persian name: *sir*)

*A. sativum* (garlic) is a traditional plant has been used as food flavour and herbal medicine for thousands of years in many countries. Different forms of extract have therapeutic effects on many different types of tumours, microbial disease, blood glucose concentration and cardiovascular disorders (37). In one study, BALB/c mice macrophages infected by *L. major* were treated by garlic extracts (38). In similar study, promastigote growth was controlled by allicin, an active ingredient in *A. sativum* (39). In another study, cell death of promastigotes occurred after exposed to *A. sativum* extract (40).

### *Camellia sinensis* (common name: *green tea*; Persian name: *Chay Sabz*)

Ethanolic extract of Green tea has higher anti-leishmania effect on promastigotes of *L. major* in comparison with glucantime (41). The active ingredient is Epigallocatechin-3-gallate (EGCG), a dietary polyphenol (flavanol). EGCG causes mitochondrial damage in *L. donovani*, *L. amazonensis, Trypanosoma rhodesiense*, *T. brucei* and *T. cruzi* (42).

### *Thymus vulgaris* (Common name: *thyme, serpyllum*: Persian name: *avishan*)

*T. vulgaris* was effective on parasitic disease such as trichomoniasis, amoebiasis, leishmaniasis, giardiasis, and toxoplasmosis (43). Fourteen species of avishan exist in Iran. Steam and fresh flower extract are used in herbal therapy. Comparison to glucantime, hydroalcoholic extract of *Achillea millefolium* is significantly more effective to reduce of leishmaniasis ulcer size (43). Apoptosis of *L. major* occurs after adding essential oil and hexane extract of *T. vulgaris* to cell culture (44, 45). Carvacrol, borneol, thymol are important bioactive components reported as anti-amoebic ingredient (45).

### *Salvia officinalis* (Common name: sage; Persian name: Maryam goli)

There are more than 750 salvias throughout the world. Fifty-eight species of *S. officinalis* are found in Iran, 17 of which are native varieties. Methanolic extracts of *S. officinalis* leaves reduces number of amastigote and promastigote of *L. major* inside the macrophages (46, 47). In addition, tannins and phenols extracted from *S. officinalis* were effective on *L. donovani* and *L. major* (48).

### *Artemisia sieberi* (Common name: *wormwood*; Persian name: *dermane*)

*A. sieberi* is grown at desert areas of Kashan province and many region of Iran. Artemisinin is an aromatic herb found in the extract of some medicinal plants such as *A. sieberi* (49). It is reported as antimalarial (*P. falciparum* and *P. vivax*) and anti-trichinellosis by artemisinin (50). *L. major* promastigote and amastigotes are very sensitive to aqueous extract of *A. sieberi* and this component decrease the number of amastigotes in macrophage cell cultures (51).

### *Lawsonia inermis* (Common name: *henna*, *Mehendi*; Persian name: *hana*)

Henna leaves, flowers, seeds, stems bark and roots are used in traditional medicine *L. inermis* is known as antiparasite and antifungal herb. It is used in infections caused by *Trypanosoma*, *Leishmania*, and *Plasmodium* species (52). Growth of *L. major* promastigotes is stopped by henna*.* However, *S. officinalis* has shown more leishmanicidal activity than *L. inermis* (53).

### *Cassia fistula* (Common name: *golden shower tree*; Persian name: *khyar chambar, kharnoub*)

*C. fistula* fruit extract is known a famous antibacterial and anti-parasitic herbal medicine (54, 55). Chauhan et al. reported *C. fistula* hexane extract has significant effect on *L. chagasi* promastigotes (56). This extract is used in the lesions of CL. It is more effective than hydroalcoholic extract of *C. fistula* (57). The efficacy of concentrated boiled extract and hydroalcoholic extract of *C. fistula* is the same. This plant can be used topically with Glucantime to lower drug dose and duration of treatment (58, 59). Studies on side effects and toxicity of this plant showed no adverse reaction after using *C. fistula* fruit extract even with higher dosages (60). Additive effect of *C. fistula* fruit extract with glucantime has been considered by several investigators (59, 60).

### *Seidlitzia rosmarinus* (Common name: *Julman, Salsola rosmarinus, shenan, Suaeda rosmarinus*; Persian name: *Eshnan*)

*S. rosmarinus* grows on the salt desert areas of the world. It is cylindrical and has fleshy leaves containing abundant minerals. It is used as forage for animals in desert areas. Leaves and stems are used in soap industry (61). This plant is natives in Khorasan Province and is used for treatment of CL lesions by native. They put pure dried leaves’ powder of *S. rosmarinus* on their skin lesions suspected to oriental sore. Hydro-alcoholic extract of *S. rosmarinus* was used with concentrations 5%, 10%, and 15% to treat experimental CL in BALB/c. Survival rate was significantly higher compared to control group (*P*=0.001) and concentrations below 15% did not show a therapeutic effect on experimental CL ulcers of BALB/c mice (62).

### *Euphorbia myrsinitis* (Common name: *spurge*; Persian name: *Farfion*)

*Euphorbia* grows all over the world. There are seventy species of herbaceous in this genus. The milky latex or sap has medical value with highly toxicity and irritation of skin and eye (63). Main active ingredients are diterpene polyesters and other terpene used as herbal medicine (64). *Euphorbia* species have been the source of a large number of biologically active compounds (65). Cytotoxicity effect (66), skin irritant (67, 68), dermatitis, conjunctivitis (69) and inflammatory reactions are biological activities of some *Euphorbia* species (70). Other effects are antioxidant, antiviral (71) and antileishmanial (72, 73). Antileishmaniacidal effect of methanol extracts of *Euphorbia lagascae* seeds has been studied on *L. donovani*, *L. infantum* promastigotes and on *L. major* and *L. donovani* amatigotes (73).

### *Satureja khuzestanica* (Persian name: *Marzeh khuzestani*)

Sixteen species of this genus have been reported from Iran (74). It is well-known for its therapeutic effects and used as antiseptic and analgesic herb. Because *Satureja* can give a large amount of essential oil, it is used in the pharmaceutical, perfumery, food and cosmetics industries (75). *Satureja* species have been used in traditional medicine as antibacterial, spasmolytic, diuretic agents and cicatrisant. *S. khuzestanica* leaves extract contain active ingredients, which could be candidate as suitable herbal drug in treatment of experimental cutaneous leishmaniasis in vivo (76) and used as leishmaniacidal agent in vitro (77, 78). Further studies would therefore be needed to see clinical response and associated toxicities in vivo.

## Discussion

“Leishmaniasis is a broad-spectrum parasitic disease reported worldwide. Until now no effective vaccine or drug for the inhibition of parasite has been reported and no effective chemicals for eradication of carriers is provided” (79).

Nowadays, efforts are continued to discover an effective route of treatment to cure CL with minimal side effects. Glucantime is used as a standard medicine, but it has many side effects like erythema, edema, local pruritus, urticarial and sometimes local swelling, nausea and vomiting, diffuse erythema and shock (80). There are still many problems in treatment of CL even by standard protocols. Traditional treatment of CL is a common habit of natives in many endemic areas.

This disease is a great health problem in Iran. The prevalence of infection has been reported as 1.8% to 37.9% in different provinces of Iran (81). There exist more than 250000 genera of medical plants in the world. More than 50 genera of them are effective in treatment of CL lesions. Almost 80% reported which working on CL treatment has used only traditional remedies (82). Many of them grow in special climate and different geographical areas of Iran that are unique in the world. The same time, you can ski at the north of Iran, wear spring coat at the center and swim in the Persian Gulf. All these plants grow in such different climate and nature of Iran. On the other hand the growth condition for each plant is unique for example *B. vulgaris* grows in south Khorasan Province but *E. myrsinites* in the north as well as in the south of Iran (83), *S. rosmarinus* grows in desert climates and this type of environment is observed in many provinces of central and south of Iran.

The question why we do not have enough traditional medicine in the pharmacies and market which we have several antileishmanial plants is noticeable. The main reason may be due to imperfect researches. The investigations on medical plants mostly performed by PhD students, therefore these researches were not continued and followed by other investigators.

According to endemicity of cutaneous leishmaniasis in Iran, we propose more clinical researches to determine the effectiveness and safety of native plants and their active ingredients, and possible toxic substances can lead to the production of efficient and safe drugs for treatment of CL. Formulation and production of ointments containing herbal extract or essential oil are noninvasive method for treatment of CL. Lack of an efficient vaccine and resistance to drugs administered for the treatment of leishmaniasis is required of preparation of herbal ointment on wound healing and finding an effective way of reducing injection pain and the treatment cost.

## Conclusion

Ttraditional treatment of CL by herbal medicine is recommended by many investigators. Efficacy and safety of some of the more promising traditional remedies used by local populations as possible future alternatives to Glucantime.
